# Left Ventricular Intussusception of an Intimal Flap in an Aortic Dissection Stanford Type A

**DOI:** 10.12945/j.aorta.2017.16.048

**Published:** 2018-09-24

**Authors:** Tim Kaufeld, Malakh Shrestha, Axel Haverich, Andreas Martens

**Affiliations:** 1 Department of Cardiothoracic, Transplant and Vascular Surgery, Hannover Medical School, Hannover, Germany

**Keywords:** Dissection, Aorta, Intimal flap, Intussusception

## Abstract

A 75-year-old woman was admitted to the emergency department with severe and sudden chest pain. Transthoracic echocardiogram showed an unusual case of aortic dissection Stanford Type A with complete circumferential detachment of the ascending aorta intima. An intussusception of the intima flap into the left ventricular outflow tract was also observed. This case presents a very rare surgical treatment involving root repair using tissue adhesives for a left ventricular intimal flap.

A 75-year-old woman was admitted to the emergency department with severe sudden chest pain, signs of malperfusion of the right arm, and left-sided hemiplegia. Transthoracic echocardiogram showed an acute aortic dissection Stanford Type A. Preoperative transesophageal echocardiography ( Video 1 ) demonstrated a complete circumferential dissection of the ascending aorta with remaining attachment in close proximity to both coronary ostia. An invagination of the complete intimal flap across the aortic valve into the left ventricle was seen during diastole ( Figure 1A ). During systole, the flap was pumped into the left ventricular outflow tract ( Figure 1B ). As a result of this intussusception, the patient showed severe aortic insufficiency. Furthermore, a dissection of the brachiocephalic trunk with occlusion of the true lumen was observed. Despite severe aortic insufficiency and repetitive occlusion of both coronary ostia, the patient showed no periods of hypotension. There was no need for inotropes or intubation.

**Video 1.** Long-axis transesophageal echocardiography showing complete circumferential detachment of the ascending aorta and intussusception of the intimal flap into the left ventricular outflow tract.

**Figure 1.**
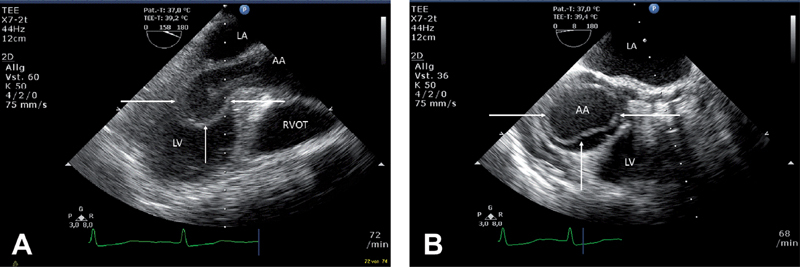
*Panel A.* Long-axis echocardiogram showing intussusception of an intimal flap into the left ventricle. *Panel B.* Short-axis echocardiogram showing circumferential dissection of the ascending aorta with the intimal flap in the left ventricular outflow tract.

Due to impending hemodynamic collapse, emergency surgery was performed. After opening the pericardium, the floating intimal membrane was visible under a transparent adventitial layer. The aortic root was repaired using tissue adhesives, and replacement of the supracommisural ascending aorta was performed. The existing intimal flap was additionally fixed with the main graft to the root suture line. Subsequently, the brachiocephalic trunk and aortic hemiarch were replaced under deep hypothermic circulatory arrest. Postoperative computed tomography scanning after surgery confirmed adequate perfusion of the supra-aortic vessels and complete occlusion of the false lumen. The patient was transferred to the admitting clinic after 4 days. Preoperatively existing aphasia improved significantly after 3 weeks of rehabilitation.

